# Embracing the European Regulation in The Netherlands: VGO implementation status, its threats and opportunities^☆^

**DOI:** 10.1016/j.conctc.2022.100957

**Published:** 2022-07-02

**Authors:** Sofia Di Martino, Rieke van der Graaf

**Affiliations:** a Utrecht University, Utrecht, the Netherlands; b Department of Medical Humanities, Julius Center for Health Sciences and Primary Care, University Medical Center Utrecht, Utrecht, the Netherlands

**Keywords:** European clinical trials regulation, The Netherlands, VGO implementation, Local feasibility, ACRON, Association of Contract Research Organizations, BoD, Board of Directors, CRO, Clinical Research Organization, CT, Clinical Trials, CTA, Clinical Trial Application, CTIS, Clinical Trial Information System, DCRF, Dutch Clinical Research Foundation, ECTR, European Clinical Trial Regulation, MS, Member State, NFU, Dutch Federation of University Medical Centres, STZ, Collaborating Top Clinical Training Hospitals, VGO, Site Suitability Declaration, VIG, Association of Innovative Medicines, WMO, Medical Research Involving Human Subjects

## Abstract

**Background:**

In 2014, the European Clinical Trials Regulation was drawn up by the European Commission to replace the Clinical Trials Directive. The new Regulation aims to solve the shortcomings revealed by the Directive, such as extensive timelines and high bureaucratic costs, while increasing standards for safety and transparency of clinical trials. Importantly, the Regulation also points at harmonizing procedures among European Member States. From January 31st, 2022, it will be possible to submit clinical studies through a new portal, namely the Clinical Trials Information System. Since not complying to the Regulation implies not participating in clinical trials, many European countries underwent changes in national documents and related procedures. In The Netherlands, the Site Suitability Declaration, a document necessary to ascertain the adequacy of a site to perform a trial, was reviewed.

**Methods:**

In our research, we investigated the status of the VGO implementation during a transition period among different stakeholders involved in the start-up process through a validated questionnaire and subsequent semi-structured interviews.

**Results:**

This project showed a slow-paced implementation, linked to communication and organizational challenges but also to a negative approach towards the change. Nevertheless, some stakeholders expressed constructive feedback as well, indicating the VGO as an upgrade. The latter was mainly achieved through establishing a trustful relationship with other stakeholders, undergoing additional adjustments, and having a positive mindset.

**Conclusions:**

This research pointed at a still too scarce collaboration between stakeholders, who should rather actively contribute to achieve the implementation goal.

## 1 Introduction

In November 2020, the Dutch Clinical Research Foundation (DCRF) released the VGO document and the Local Feasibility Procedure [[1], [2]]. The document is the new Site Suitability Declaration for any Dutch healthcare institution to ascertain their suitability and capacity of conducting a clinical trial [3]. Clinical trials (CT) are a type of clinical studies, also named interventional studies, where an investigation on medicinal products occurs on humans to ascertaining their safety and/or efficacy [3].

This national documentation change arose from a modification of the European regulatory framework, transitioning from the Clinical Trial Directive to the European Clinical Trials Regulation (ECTR 536/2014). The ECTR aims to ameliorate the current situation, since with the Directive into place between 2007 and 2011, the authorization of CT registered a 25% decrease, with an average time of 152 days and high costs [4]. Moreover, a highly competitive environment existed within European countries since countries with shorter timelines and lower costs were more likely to be selected. The ECTR focuses on making the CT authorization process simpler and centralized, and accelerate the current pace of submission, approval and start-up [3].

The VGO is comprised of two parts [2]. Part A states the suitability and feasibility of the clinical center for study participation, while Part B contains more detailed information about the study, where various appendices describe the departments’ involvement, with all the necessary research operations depiction. Part B needs to be signed by each head of the department involved to ascertain the feasibility, while Part A is directly assigned to the Board of Directors to be reviewed. Besides, the major target of the new Feasibility Procedure is to ensure having the first trial participant in as soon as possible after dossier approval and stick to the timelines imposed by the ECTR [1].

One of the main novelties introduced along with the ECTR is the Clinical Trials Information System (CTIS) web portal, a single entry point to submit CT applications [5]. This new platform favors multi-country CT since the outcome is a single assessment, where all the countries involved will receive simultaneous approval within three months from the study submission. Each application is comprised of two parts [6]. Part 1 concerns the technical, scientific, non-clinical and clinical quality and it is reviewed jointly by the National Authorities of each Member State (MS), guided by one MS chosen by the sponsor, namely the reporting Member State [7]. The assessment of Part 2 is undertaken by every MS involved, since it regards local feasibility and ethical aspects as well as local patient information documentation, which can vary among MSs. Part A of the Dutch Site Suitability Declaration represents an integral part of Part 2 of the study dossier. Undoubtfully, the Regulation states short and precise timelines, having a maximum of 106 days from submission to approval. The two dossier Parts could be assessed simultaneously at applicant discretion [3,8].

Since implementing the VGO in The Netherlands is crucial to comply with the new European Regulation, this research project was conceived to understand the status of its implementation among different stakeholders involved in the local feasibility process before the VGO officially comes into force (transition phase). Of utmost importance is gaining insights into the hurdles stakeholders are experiencing and then bringing forward feasible solutions to ease the transition. Since no studies have been conducted on this topic yet, this article provides a baseline for future research.

## 2 Methods

The stated aim was pursued via mixed methods research. A sequential explanatory model was chosen, where a questionnaire and one-to-one in-depth interviews were conducted (QUAN→QUAL). The questionnaire and interviews were designed on the theoretical background to address the research questions.

Given the national relevance in understanding the voluntary implementation status of the VGO during the transition phase, the DCRF joined the project providing feedback and validating the questionnaire. 16 closed-ended multiple choice questions were drawn up addressing the participant's role in the process, their experience and knowledge of the VGO, procedure and related timelines and were asked to provide suggestions. The questionnaire was released on March 15th, 2021, via Google forms and shared for recruitment via the DCRF contact list and newsletter. Participation was voluntary. The completion of the questionnaire took approximately 10 min, and the link was active for 15 working days. The final product in English can be found in the Supplemental material (*S1*).

As part of the questionnaire, we invited the participants to take part in the interviews. An heterogenous group of people was selected for the interviews: 13 people with experience with the VGO, 7 without and 1 as member of the DCRF. This qualitative research was designed to deepen the conversation about the VGO and further explore the reasons behind the participants’ answers of the questionnaire. The interviews lasted 30–45 min and were recorded via Zoom. To better conduct the interviews, a semi-structured guide was exploited and can be found as Supplemental material (*S2*). A particular focus was on acknowledging experiences, understanding the practical implications of the implementation, testing awareness on the context and investigate the timelines feasibility. Considering the different categories of stakeholders involved, the questions were slightly adapted to fit the informant function. At the end of each interview, suggestions on the document and related procedure were asked. Importantly, the guide evolved throughout the process since the interim analysis revealed saturation of some questions. This development aimed to further explore some aspects raised in previous interviews and leaded to a deeper knowledge of the topic.

### 2.1 Data analysis

Upon the closure of the questionnaire, the answers were downloaded in an Excel file, categorized based on the working group and analyzed. For the analysis, the Excel function “COUNTIF” was used. When the results needed further processing, these were encoded through the qualitative data analysis software *NVIVO12* , which functioning is explained in detail in the following paragraph.

Shortly after each interview, the conversation was transcribed verbatim into a text document file. The files were uploaded into the software program *NVIVO12* to proceed with the thematic analysis [9]. SDM independently coded the transcripts subsequently performing open, axial, and selective coding. When higher order themes were reached, the quality of the work was assessed firstly through team meetings and then through member validation by two different stakeholders (one from an institution and one from a sponsor).

## 3 Results

### 3.1 Questionnaire findings

222 people were directly reached by an email containing the link to the questionnaire, of which 113 completed it. Based on questions 1 and 2, their responses were divided in the following categories: “VIG/ACRON CRO” (*VIG = Association of Innovative Medicines; ACRON = Association of Contract Research Organizations*; *CRO = Clinical Research Organization*), “hospital research professionals”, “hospital research commission”, “hospital departments” and “others” (MREC members, quality assurance and CMC personnel and financial advisors). The number of responses per category was 30, 43, 22, 8 and 10, respectively.

**Fig. 1:**
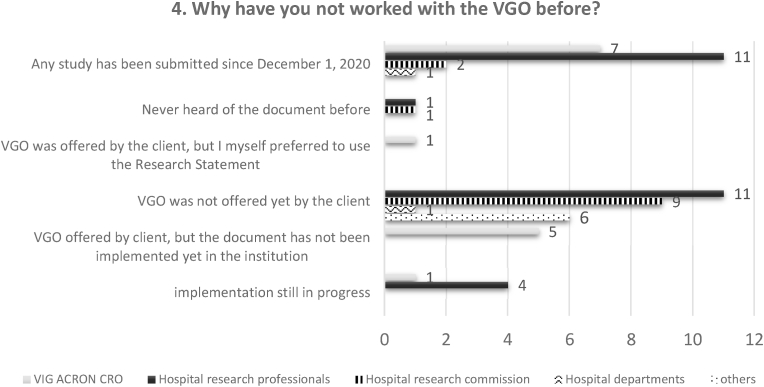
The plethora of reasons behind the failing implementation. This graph shows the responses to question 4 “Why have you not worked with the VGO so far?“. It is possible to appreciate the various reasons of different categories of stakeholders for not implementing the document. Excluding who have not done studies recently and for whom the implementation is still in progress, the VGO was either not accepted by or not offered to the site.

Most stakeholders had not implemented the VGO in the transition phase (72 people, 63% of the total). The reasons behind their input were various, where the most upvoted was “VGO was not offered by the sponsor” (Fig. 1). This was mainly chosen by hospital research professionals, research commissions and ‘others’, while the majority of CRO personnel indicated that the VGO was either not accepted by the institution or they have not done studies since the VGO release. 32% of stakeholders had experience with the document. However, in total, the VGO was used in 55 studies out of 142 (38%). While CRO employees and research commission members used the document for roughly 50% of their studies, the other categories for 30%. Looking at the uptake of this document 4 months later, we saw a still low usage. Among those with experience, 96% used both Part A and Part B of the template.

**Fig. 2:**
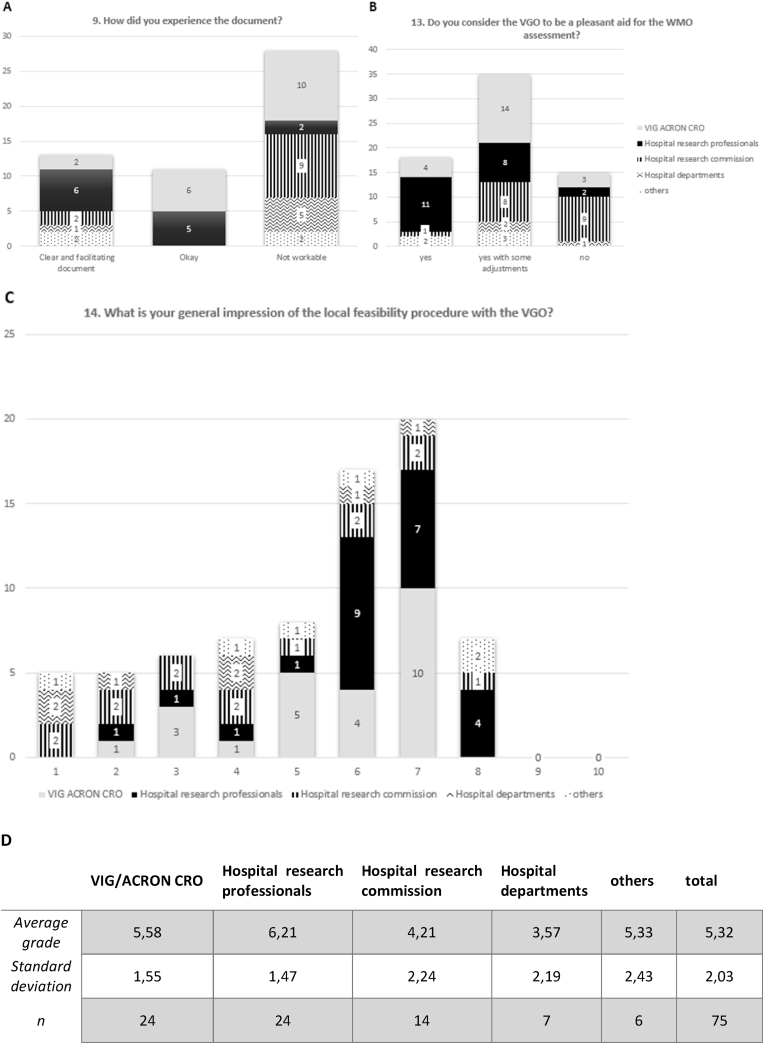
The feedback was discordant throughout the questions and heterogenous within the same working category. **A** Question 9 regarded the experience with the VGO and found most of respondents defining it as ‘not workable’ (56%). The rest marked it ‘okay’ or ‘clear and facilitating’. **B** This graph shows how many people found pleasant working with the new document. 78% expressed a positive opinion but highlighting the need for improvements. **C** In this graph, the grades given to the procedure asked in question 14 are reported. **D** This table reports averages and standard deviations of the grades given by the different categories to the feasibility procedure with the respective n number (data from question 14).

Throughout the questionnaire, the feedback on the document and procedure was asked thrice in different modalities (Fig. 2). When asking about the participants' experience with the VGO, 54% of the respondents chose ‘not workable’ to define it, while 21% described it as ‘okay’ and 25% as ‘clear and facilitating’ (the last two were considered as positive feedback). None of the categories gave a unanimous positive or negative feedback, but hospital research commissions and hospital department personnel mostly provided a negative one, while hospital research professionals a positive one (Fig. 2A). More towards the end of the questionnaire stakeholders were asked whether the VGO could be considered a pleasant aid, where 78% voted for either ‘yes’ or ‘yes with some adjustments’ (Fig. 2B). No category fully voted for ‘no’, even though half of the research commission members did. Lastly, a general impression on the local feasibility procedure was recorded, where participants voted with score ranging from 1 to 10 (with 10 being the highest rank). 59% gave a sufficient grade, from 6 to 8, while the remaining 41% assigned an insufficient mark (1–5) (Fig. 2C). Taken together, these data show discordance among the questions and heterogeneity within the same working category when giving feedback.

**Fig. 3:**
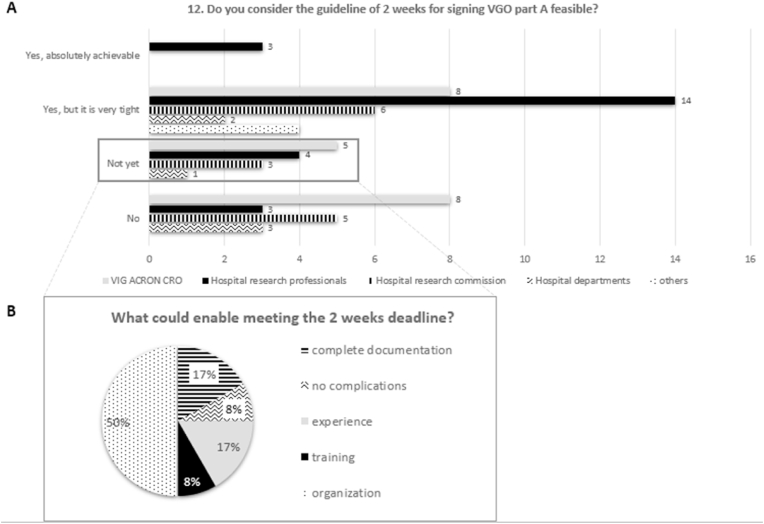
Timelines feasibility and where to improve. **A** The clustered bar depicts the different answers given to question 12. The answers indicated that 50% found the deadlines tight but feasible, 4% completely achievable while 27% impossible to meet. The remaining 19% expressed that these timelines are not yet possible. **B** This chart shows the coded-driven analysis of the answer ‘not yet’ presented in Fig.3A, where 50% of respondents pinpointed a necessary organizational improvement.

The DCRF established a deadline of 2 weeks for having the VGO signed back by institutions. Investigating the experience with the deadline, 42% sticked to it, for the 21% took from 3 to 4 weeks, while for the 24% more than 6 weeks. Besides, 50% of the stakeholders described this deadline as tight but achievable, whereas only 4% felt complete certain on their feasibility (Fig. 3A). The rest reported that 2-weeks are not enough (27%) or that this guideline is not yet achievable. The latter category was encoded to understand what could enable meeting the deadline. Fig. 3B shows that solving organizational issues is crucial to fulfil this requirement (50%), while the other half suggested receiving a more complete documentation, having no complications within the study protocol, undergoing more training, or getting more experience.

We identified some misunderstanding over two concepts. When inquiring into which Part of the VGO goes in the submission file, only 61,5% gave the correct answer (only Part A), while 33,3% voted both Part A&B and 5,2% Part B. Moreover, the responsibility for the completion of the form appeared still unclear, since only 58% allocated it to the sponsor.

Lastly, the participants were invited to leave one or more suggestions to improve the VGO and/or related procedure. 51% of the responses regarded tips ‘on the document itself’, suggesting providing more clear information within the form, changing its format, and making it more editable. 33% would like to have external aids, like more clear communication, improve organizational aspects, undergo trainings, and support the VGO with extra documentation when asked. The remaining entries regarded harsh comments about the 2-weeks deadline or highlighted the unworkability of the new form. Many of these suggestions were similarly found in the qualitative part of the research and they are reported in the next sections.

### 3.2 Interviews’ findings

**Fig. 4:**
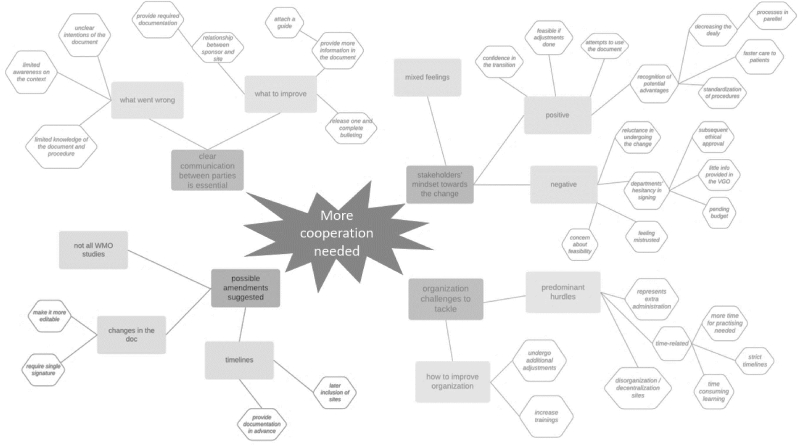
Mind map of the selective coding. Results of the interviews' spiral of analysis presented through a mind map. In the center, the core category ‘more cooperation needed’ is reported, surrounded by four main themes.

In total, 21 interviews were done in a timeframe of a month upon the closure of the questionnaire. We defined one core category and four main themes (Fig. 4).

One of the main themes regarding the need for clear communication. This topic was touched upon by almost all interviewees and expressed the lack of communication between different stakeholders involved in the local feasibility procedure. Unclarity was found regarding the intentions of the document and the ECTR context. In general, the VGO is perceived as more binding than it actually is. The following quote reports the words of a CRO employee explaining this concept based on their experience:

“It is not like they are hooked at the VGO signature [the institutions]. They are not stuck with the study, if for some reason the final contract with the board of directors is not signed, then we can toss away the VGO, it is just a sort of commitment that they can do it. […] It is more an intention based on what it is known now, like “we have the resources and the facilities to participate”.”

Besides, stakeholders proposed several ways to improve communication. They expressed the need to receive a clear and complete bulletin, summarizing all important and definitive information. Moreover, also adding information within the document itself could clarify some points. Another approach suggested to improve the communication is ameliorating the relationship between the sponsor and the sites. Table 1 reports some quotations which are useful to understand the points of view of the different stakeholders. Different people among sites were found declaring to suffer from the little contact with the sponsor, while others highlighted a resistance from the sponsor to provide the asked documentation (Table 1^*a, b*^). We could establish that requesting more documentation derived from the fear of signing the VGO without having a clear overview and it could be linked to the misperception of the form's legal bond reported before. Considering the sponsor side, we identified two main reasons behind this resistance: not having received manuals from corporate yet or thinking that providing extra material will increase the site's revision time. For CRO employees, developing a trustful relationship between trials co-workers is essential (Table 1^*c, d*^).

**Table 1:** Table reporting quotes from different stakeholders regarding the relationship between the sponsor and the sites. Some quotations were slightly modified to enhance readability.

Interviews quotations encoded in ‘relationship between sponsor and sites’	
NFU pharmacist^a^	*With the old procedure we were in direct contact with the sponsor through the CRA, while now we are not in contact anymore. So, it feels more chaotic, because we need to make assumptions sometimes, and in one case the sponsor contacted us asking for something we already had sent in. I am really missing the direct contact.* *[…] we can do a lot of studies, but we need some guidance.*
STZ coordinator^b^	*I think that the 2 weeks could be feasible, but it also depends on the sponsor side, so it won't be on us only. Talking with the sponsor it seems not so easy to have all the documentation needed at the right moment, like the manuals, so it would be difficult in that case, it is dependent on how good info is provided. Only the protocol is not enough, the departments need more information. The more info we have the better it is for the timelines.* *[*…*] it is crucial to receive all the important information from the sponsor side to have a clear communication and undergo a correct feasibility process.*
CRO employee 1^c^	*I think what we are trying to elaborate is how we can be of help. How we can actually help for instance physicians. So many people are willing to help to prepare them. There is no need for them to learn all alone. We should focus on how to provide them with the information they miss.* *[…] I think that if there is a base of trust between the sponsor and the site it will save lot of time on both sides, would be better for patients as well.* *They should not insist on doing things as before.*
CRO employee 2^d^	*I think the departments have their own way of working. I saw that the contracts for the studies, especially the radiology, have their own culture, most of the times they are refusing to sign, it is even hard to discuss the budget* *Interviewer: so, is there reluctance in trusting the CRO/pharma?* *Yes*

NFU: Dutch Federation of University Medical Centers; STZ: Collaborating Top Clinical Training Hospitals; CRO: Clinical Research Organization.

We identified three main challenges with the new procedure organization-wise. The biggest was time-related since the new way has strict timelines, a time-consuming learning process, and the time to practice was little. Moreover, some stakeholders considered it as extra administration, having to ascertain the feasibility twice (with the VGO and when reviewing the CTA). Lastly, during some interviews we realized and concluded that the decentralization of academic hospitals does not help with the transition and could entail all mentioned challenges. Below some examples from the interviews reporting these decentralization issues.

Interviewer: do you think your difficulty in meeting the timelines is because of the decentralization of the hospital?

NFU employee: this is the case for most hospitals, there is no central person for submission or approval of protocols. It is very decentralized, own dept with own budget. we need to wait for the approval by each department … It is really decentralized

_______________________________________

Interviewer: how are you experiencing the 2-weeks’ timeline?

CRO employee: it is short, but not from our side, we need to be staffed to make the VGO document, to have the information, to make the document in which the site can decide, there is pressure on that. The site has 2 weeks to sign, I cannot speak for them, but they need to change the internal processes as well. Since now it takes weeks, and it is not up to us.

Clearly, some interviewees did not experience remarkable issues when implementing the VGO and also defined the transition as an upgrade. To achieve this goal, a hospital coordinator defined a study-dedicated person, whose responsibilities were inter-department communication and being up to date with information from the DCRF. To shorten the time needed to assess the form, another coordinator thought about listing all possible procedures for each department, enabling a simple cross-check for upcoming studies. Furthermore, some sites organized meetings to raise awareness on the ECTR context and set up dedicated trainings.

The VGO transition was mainly approached in two ways: interviewees who showed a positive mindset towards this change and who did the opposite (Table 2). This theme emerged consistently within and across all interviews and memos. Stakeholders voiced their doubts over the procedure, questioning its feasibility in the given timelines and modalities, and showing a marked reluctance in undergoing the change for an abundance of reasons. According to an heterogenous group of interviewees, the feeling of mistrust between stakeholders represented a great hindrance during the implementation attempts. Hospital departments seemed the most affected by this shift and we identified three arguments underlying their hesitancy in signing the document: the department feasibility cannot be assessed holding only the study protocol, the pending budget represented a serious threat to signing, and the subsequent ethical review could lead to amendments in the protocol, jeopardizing months of preparation.

**Table 2:** Table reporting quotes from different stakeholders to underline the various opinions on the implementation. The first two report a negative statement on the VGO, encoded in the ‘concern about feasibility’ node, while the third and forth a positive one, found in ‘confident in the transition’ node. Some quotations were sometimes slightly modified to enhance readability.

*Interviews quotations encoded in ‘*stakeholders’ mindset towards the change’	
NFU study coordinator	Interviewer: do you think is it more about having more training with the document or will it never work fine? it would never work fine.
STZ study coordinator	Interviewer: and how do you feel like for the implementation? at this moment I am very much concerned about the implementation Interviewer: from your site or in general? well in general, but also given the fact that the experience we had was a little disaster because information was denied. If that is the case in general, we really have a problem, and also the timelines are of concern, but we can work on that.
NFU study coordinator	*Interviewer: are you confident in the implementation from July?* *yeah, we will manage. There will be several trainings of personnel to work well with it. I am confident we can make it up in a nice way.*
General hospital study coordinator	*for my character I like to have things really clear, so this project is exhausting [laughing]. I am challenged, but well I am confident it will work out with the help of my team.*

NFU: Dutch Federation of University Medical Centers; STZ: Collaborating Top Clinical Training Hospitals.

Interviewees shared positive and constructive comments as well. Questioning their feelings on the implementation, some stakeholders described themselves as confident in the transition, while others said that the procedure could be feasible if some adjustments are done. Moreover, three quarters of participants recognized the advantages of the new procedure, as faster care for patients and the standardization of internal procedures. Clearly, the latter was mainly appreciated by sponsors, while some sites were still struggling in the transition at the time of the interview. Looking into the status of the implementation perceived through the interviews, we counted several attempts to use the document. Even though not all were successful, these data show willingness to implement the document and try out the procedure.

All stakeholders had suggestions for the document and/or procedure. Some would like to have the VGO amended basically in two ways: making it more editable and requiring a single signature. The latter regards the current requirement for a Board of Directors’ member signature on Part A and for each head of departments on Part B. Thus, some people advocated that the BoD should be responsible for both Parts. Another hot topic were the timelines. To meet them, stakeholders suggested to anticipate the provision of the documentation necessary for filing the suitability declaration. In this way, the timelines could be extended still complying to the Regulation and more importantly without having a delayed patientcare. Lastly, few stakeholders mentioned their struggle in using the document for all studies involving human subjects (WMO). This quote clearly presents the issue encountered in an academic hospital.

“If it will be only for drug related studies, we can adapt to it, but it is now the case of all the studies. […] We can have 150/200 studies approved each year, but only 50 are drug related. At the moment it feels impossible since it is not standard practice yet.”

Given the large number of studies, it would be challenging to apply the VGO to all of them; thus, using it only for interventional studies (CTIS-related) could be a suitable option.

Through the selective coding, we identified the core category of our findings: enhancing the cooperation (Fig. 4). This was found connected to all main themes. In fact, to improve the organizational aspects, have a clearer communication and approach the change positively, a higher level of cooperation was reported as necessary by different stakeholders. This entails the mentioned sponsor - site relationship, where improvements are essential at an organizational level, as planning more ahead and using a common strategy, but also through a more open, clear, and frequent communication. Moreover, more cooperation was requested from the DCRF as well since it should have provided more assistance during the implementation.

## 4 Discussion

There was a common wish from the DCRF and Health Authorities to set a baseline for the VGO implementation status in Spring 2021. Through our research, we found out that the stakeholders involved in the process are moving towards the VGO implementation, but the pace appears still too slow. In fact, only the 32% of stakeholders had worked with the VGO up to our research timepoint. Although the implementation is still voluntary, this finding causes concern when looking at the approaching CTIS go-live date set on January 31st, 2021. Besides, investigating the stakeholders’ point of view on the document and procedure, we identified differences between working categories and also between hospital types. Unfortunately, due to the lack of published data on this topic, it was not possible to interpret our findings in light of previous evidence.

We linked the slow-paced implementation to several reasons. For instance, the choice of having VGO Part B replacing in-site paperwork brought to an incredible resistance from groups of stakeholders. Especially department personnel and research commission members mostly shared negative feedback since the new procedure negatively interfered in their way of working. Nevertheless, we did see the advantage of having Part B as well. The standardization of internal procedures brings a positive change to sponsor employees and research professionals which will have the same file type for every study and would make the budget drawn up easier. Concerning the sites perspective on this choice, the feedback was heterogenous, but we noticed that it was more related to the hospital type than to the job role. Academic hospitals work in a decentralized way and therefore they experienced more difficulties when working with the VGO since it requires an advanced cooperation between the departments and a responsible person to act as intermediate. This professional role was hardly found in academic hospitals, while in specialized hospitals and general hospitals this professional figure had an important function in integrating the new process. We linked the presence of this figure in these hospitals to the lack of tremendous hurdles when implementing the VGO.

While we do recognize the advantages of the form as it was drawn up (Part A&B), we saw its shortcomings as well. For instance, the timeframe from the VGO publishment in November 2020 to the envisioned mandatory phase (June 2021) was not long enough and, considering the 2-weeks deadline, it leaded to a great deal of time pressure. Moreover, lagging communication brought consistent misunderstanding on various aspects of the new way of working, especially on the intentions of the document and awareness on the ECTR context.

Among various, crucial aspects necessary to boost the implementation are raising awareness on the context of the ECTR and on the consequences of not complying to it, having a positive mindset towards the change, define a person who is directly responsible for the study in the hospital and develop a better network between hospitals. Moreover, it is essential to have efficient collaboration between stakeholders. In fact, participants pointed at the lack of cooperation, since the implementation is not seen as a shared goal but rather as something unpleasant and mandatory to do. This hesitancy in working together stems from a lack of trust, which is predominant and a major hurdle to tackle.

Our research presented some limitations. For the questionnaire, we could not verify from which hospital or institution the participants were, therefore, the sample might have been too narrow. Moreover, only women participated in the interviews. We could have not prevented this anyhow since gender was not asked within the questionnaire. However, the interviews did not focus on gender-sensitive topics and therefore we remain confident the findings would have not deviated consistently if men took part. No Board of Directors members wanted to be interviewed and the department personnel sample was smaller than we hoped for. The latter represents one of the major drawbacks of this study since this category provided particular negative feedback in the questionnaire. In fact, it would have been interesting to verify if the responses had stayed similar expanding this group or if we had just caught the negative responders. Moreover, we could not clearly identify why research professionals and research commission members expressed such different feedback, positive and negative, respectively. Diving deeper into their reasons could have pointed at a more precise diagnosis of underlying issues. Therefore, future studies should take more care in participants sampling.

Our findings shared with the DCRF in May 2021 contributed to identify the learning curve of implementation and the need for more trainings. Therefore, they decided to develop a VGO education module and added a Q&A section on their website. Furthermore, a new version of the document was drawn up and released July 1st, 2021 [10]. The VGO itself was amended enhancing the visibility of the instructions and explanations, and a disclaimer about completing the document having the study protocol only has been added. However, the biggest variations were the Annex Part B ‘Staff workload department local principal investigator’ complete makeover, and the removal of the required signature of each head of departments in Part B annexes. Lastly, its mandatory use was moved to November 1st, 2021, and for industry sponsored interventional studies first.

Future research investigating the status of implementation of this document should try to understand whether the document amendments, the provided training and enhanced communication improved its workability. To yield these insights, it is crucial to ask whether progresses have been made in meeting the two-weeks deadline, in their relationship with other stakeholders involved, in ameliorating organizational aspects, and in their mindset.

## 5 Conclusions

To conclude, this project provides a baseline on the VGO implementation status in Spring 2021, defining it as slow-paced. This was linked to scarce collaboration between stakeholders involved in the process, which brought to organizational challenges within the facilities, significant misunderstandings on different aspects both at a sponsor and site level, and reluctance in undergoing the change. Nevertheless, some stakeholders were able to implement the document in the correct manner and this was mainly done having a positive mindset towards it, undergoing organizational adjustments, and establishing a good relationship with the counterpart. Undoubtfully, our data show that there is some room for improvement in the VGO, but they also reveal that there is room for change. Our suggestions were submitted to the DCRF and resulted in a new version of the document published July 1st, 2021. Looking at the consequences of not complying to the ECTR, an active contribution from all parties involved is of utmost importance to achieve the implementation goal and make sure The Netherlands remains an attractive partner for performing clinical trials and guarantee patients’ access to new drugs and therapies.

## Declaration of competing interest

The authors declare that they have no known competing financial interests or personal relationships that could have appeared to influence the work reported in this paper.
