# Supplementary material for: Embracing the European Regulation in The Netherlands: VGO implementation status, its threats and opportunities
Source: Contemp Clin Trials Commun. 2022 Jul 2;28:100957. doi: 10.1016/j.conctc.2022.100957 (PMC9287159; doi:10.1016/j.conctc.2022.100957)
Supplement: Multimedia component 1 [file mmc1.docx]

Embracing the European Regulation in The Netherlands: VGO implementation status, its threats and opportunities

VGO implementation status in light of the ECTR

Sofia Di Martino MSc ^a^, Rieke van der Graaf PhD ^b^

a Utrecht University, Utrecht, The Netherlands

b Department of Medical Humanities, Julius Center for Health Sciences and Primary Care, University Medical Center Utrecht, Utrecht, The Netherlands


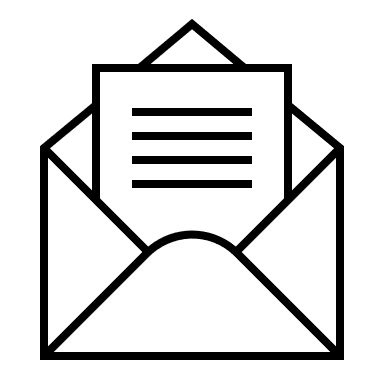
 Correspondent author: Sofia Di Martino, [dimartinosofia97@gmail.com](mailto:dimartinosofia97@gmail.com), +39 3466329516

The authors would like to thank the members of VGO working group within the Dutch Clinical Research Foundation (DCRF) for sharing their insights on the issues raised in this paper and for the validation of the questionnaire used as research methodology.

# Supplemental material

## S1: English version of the submitted questionnaire

Best,

Since the publication of the *Declaration of Suitability for Research Institutes* (VGO) last November by the CCMO, the submission document has stirred up a lot in clinical research. From the DCRF working group Local feasibility, we are curious about who has already worked with the document during the current transition period and what the experiences are. This is why we ask you to complete this survey. The results of this evaluation will be included in the new RET guideline of the CCMO and by the DCRF in its communication with the field.

Thank you in advance for your contribution.

SECTION 1

**Question 1. What type of organization do you work for?**

- Clinical Research Organization (ACRON)
- Pharmaceutical company (VIG)
- MRECs
- STZ hospital
- STZ hospital
- General hospital

**Question 2. What role do you have in the submission process?**

- Employee Sponsor / CRO
- Principal Investigator, responsible for submission in CTIS portal
- Researcher / research professional
- Employee science bureau / research committee
- Employee in hospital department (including lab, pharmacy, radiology, etc.)
- Other …

**Question 3 Have you ever worked with the VGO for a METC submission?**

- No - go to question 4
- Yes - go to question 5
- Not applicable (I am not involved in this process)

**Question 4. Why have you not worked with the VGO before?**

- Never heard of the document before
- Any study has been submitted since December 1, 2020
- VGO was not offered yet by the client
- VGO was offered by the client, but I myself preferred to use the Research Statement
- VGO offered by client, but the document has not been implemented yet in own institution
- Other ...

**Question 5. How many studies have you submitted since December 1, 2020, and for how many studies have you used the VGO?**

*Could you answer the question in the following format: ”10/5”?*

- ……….

**Question 6. Which parts of the VGO template did you use?**

- Part A and Part B
- Only Part A

**Question 7. What part of the VGO do you think is part of the submission file?**

- VGO part A and part B
- VGO part A only
- VGO part B only

**Question 8. After METC approval, was the study immediately opened for patient inclusion?**

- Yes, no delay
- No, because initiation visit still had to take place
- No, the departments involved were not yet ready
- No, because the contract has not yet been signed
- Other ...

**Question 9. How did you experience the document?**

- Clear and facilitating document
- Okay
- As not workable

**Question 10. Was the VGO expected to be completed (completely and in detail) by the sponsor?**

- Yes
- No

**Question 11. Within how much time was VGO part A signed by the (mandated) Board of Directors after delivery by the sponsor?**

- 2 weeks
- 3 - 4 weeks
- 5 - 6 weeks
- More than 6 weeks

**Question 12. Do you consider the guideline of 2 weeks for signing VGO part A feasible?**

if your answer is no, please enter the reason under "other".

- Yes, absolutely achievable
- Yes, but it is very tight
- Other…

**Question 13. Do you consider the VGO to be a pleasant aid for the WMO assessment?**

if your answer is no, please enter the reason under "other"

- Yes
- Yes, with some adjustments
- Other…

**Question 14. What is your general impression of the local feasibility procedure with the VGO?**


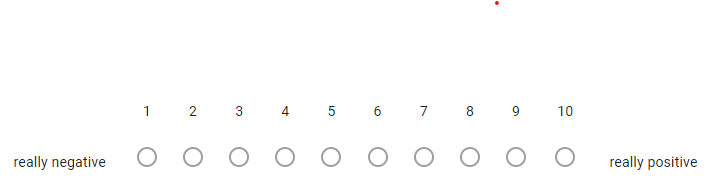


**Question 15. If you have also used the contract with VGO paragraph - has this led to questions / problems?**

if your answer is yes, please enter the reason under "other"

- No
- Contract not applicable
- Other…

**Question 16. Do you have suggestions for improvement or tips for the process with the VGO?**

- No
- Other…

SECTION 2:

**Would you also like to participate in a more in-depth interview with a student from Utrecht University?**

Deliberately, the motivation behind your answers was not asked.

An English-speaking master's student from Utrecht University, Sofia Di Martino, would like to collect in-depth opinions and experiences about the VGO as part of her graduation project at Sanofi. This is done through a one-on-one interview of 30 to 60 minutes. The interview is an opportunity to contribute to addressing current challenges that you / your organization faces during the implementation of the VGO / ECTR. The aim in this internship is to find out whether the Netherlands is ready to implement the VGO, how to tackle jointly raised issues, and to put forward feasible solutions.

In August Sofia will also share her blinded graduation report with the DCRF. If you would like to receive a blinded copy (AVG guideline), please contact her or the DCRF.

Participation is voluntary and much appreciated by both Sofia and the DCRF. If you are interested in being contacted for the interview, please enter your email address here.

**Question 17. If you are interested in being contacted for the interview, please enter your email address here.**

……………………………………………………………………….

**Question 18. If you would like to receive a copy of the final report, please enter your email address.**

……………………………………………………………………….

SECTION 3:

How do we protect your privacy?

To protect your privacy, we give a code to your data. We keep the key to the code in a safe place at Genzyme Europe B.V in The Netherlands, in the Clinical Study unit.

When we process your data, we always use that code. Even in reports and publications about the study, nobody will be able to see that it was about you.

Your personal data if provided to us (Name, affiliate, address, telephone and/or email address) will be stored until the completion of the study (September 2021) and then removed.

If you have any complaints about the processing of your personal data, please contact our DPO at: privacy.nl@sanofi.com

If you have any additional questions or you want to receive the full Data Privacy Notice, please do not hesitate to contact me at [sofia.dimartino@sanofi.com](mailto:sofia.dimartino@sanofi.com)

By continuing to complete the questionnaire, you hereby consent to the processing of the received data.

- I understand and I agree

## S2: Interviews’ guide

| Interview guide: DCRF member | |
| --- | --- |
| Thematic area 1: VGO experience | |
|  | What were common loopholes experienced pre-VGO? |
|  | How does the VGO intend to tackle these? |
|  | After the first months of VGO, how is the situation? |
|  | Did you already experience issues/restrictions with the new regulation? |
|  | Could you name 2 benefits/ and 2 difficulties that already come up? |
| Thematic area 2: VGO awareness | |
|  | What are your thoughts about the current position of NL in clinical research compared to other countries? |
|  | Has been done a process to raise awareness inside hospitals before presenting the VGO? |
|  | How would you comment on your work (as organization) in this process? |
| Thematic area 3: VGO/ECTR timelines | |
|  | What is your opinion on these timelines regarding their feasibility? |
|  | What is your expectation on the VGO implementation from July 2021? And after CTIS in December? |
|  | |
| Interview guide: sponsor and principal investigator | |
| Thematic area 1: VGO experience | |
|  | Could you please elaborate on your experience with the VGO? |
|  | Which are the most important improvements/drawbacks? |
|  | Additional questions on peculiar/outlier answers in the questionnaire |
| Thematic area 2: VGO awareness | |
|  | How did you become aware of the VGO? |
|  | Do you feel having enough theoretical and practical information for its implementation? |
|  | How has the VGO changed your job? And how the new ECTR will? |
| Thematic area 3: VGO/ECTR timelines | |
|  | How is your experience so far with these deadlines? |
|  | What is your opinion on the ECTR deadlines and in general on the process (pro or con/ feasible or not)? |
|  |  |
| Interview guide: hospital personnel | |
| Thematic area 1: VGO experience | |
|  | Could you please elaborate on your experience with the VGO? |
|  | Which are the most important improvements/drawbacks? |
|  | Has your undergone taken additional adjustments aside the VGO or is planning to do so before the ECTR? |
|  | Additional questions on peculiar/outlier answers in the questionnaire |
| Thematic area 2: VGO awareness | |
|  | How did you become aware of the VGO? |
|  | Do you feel having enough theoretical and practical information for its implementation? |
|  | How has the VGO changed your job? And how the new ECTR will? |
| Thematic area 3: VGO/ECTR timelines | |
|  | How is your experience so far with these deadlines? |
|  | What is your opinion on the ECTR deadlines and in general on the process (pro or con/ feasible or not)? |
|  |  |
| Interview guide: No experience with the VGO | |
|  | Could you elaborate on why the VGO was not implemented yet? |
| Possible follow up questions: | |
|  | Will you consider trying the document/procedure before July (when it will be mandatory?) |
|  | What do you think about its deadlines? |
|  | So, do you think a different process of awareness could have encourage you more? |
|  |  |
| At the end of all interviews | |
|  | Do you have any additional remark or suggestions? |
|  | Would you like to make further comments outside what asked in the interview? |
